# Validity and Reliability of the Facility List Coder, a New Tool to Evaluate Community Food Environments

**DOI:** 10.3390/ijerph16193578

**Published:** 2019-09-25

**Authors:** Ana María Arcila-Agudelo, Juan Carlos Muñoz-Mora, Andreu Farran-Codina

**Affiliations:** 1 Department of Nutrition, Food Science, and Gastronomy, XaRTA–INSA, Faculty of Pharmacy, University of Barcelona, Av. Prat de la Riba, Campus de l’Alimentació de Torribera, 171, Santa Coloma de Gramenet, E-08921 Barcelona, Spain; anamariaarcila1@gmail.com; 2 Department of Economics, Universidad EAFIT, Carrera 49, N 7 sur 50, Medellín 050024, Antioquia, Colombia; jmunozm1@eafit.edu.co

**Keywords:** community food environment, nutrition environment, geographical information systems (GIS), Facility List Coder, Python

## Abstract

A community food environment plays an essential role in explaining the healthy lifestyle patterns of its community members. However, there is a lack of compelling quantitative approaches to evaluate these environments. This study introduces and validates a new tool named the facility list coder (FLC), whose purpose is to assess food environments based on data sources and classification algorithms. Using the case of Mataró (Spain), we randomly selected 301 grids areas (100 m^2^), in which we conducted street audits in order to physically identify all the facilities by name, address, and type. Then, audit-identified facilities were matched with those automatically-identified and were classified using the FLC to determine its quality. Our results suggest that automatically-identified and audit-identified food environments have a high level of agreement. The intra-class correlation coefficient (ICC) estimates and their respective 95% confidence intervals for the overall sample yield the result “excellent” (ICC ≥ 0.9) for the level of reliability of the FLC.

## 1. Introduction

There is growing interest in understanding how the physical environment affects health outcomes, either directly or by creating a context in which people make health-related decisions [1]. Among the various different environs (e.g., sports facilities), community food environments have received increasing attention in the public health sector and from policy makers owing to their effects on diet and health outcomes such as obesity [2]. The transformation of the food and nutrition industry during the last decade, the increase of the availability of high calorie food (e.g., fast-food) (availability), the relative increase of healthy food prices over less healthy food options (affordability), and the increase of areas without a store where it is possible to buy fresh food (i.e., food desert) (accessibility), among other factors, evidence the fact that community food environments have changed dramatically during the last decades and play an important role in changing the food behaviors of adults as well as children [2,3,4]. 

Despite much qualitative evidence showing the influence of these new community food environments on food behaviors and health outcomes such as obesity, quantitative studies have found counter-intuitive or inconsistent results that suggest that the relationship between food environments and eating patterns is still far from being understood [2,4,5]. In a recent systematic review of the relationship between local food environments and obesity [3], they found limited evidence of the existence of this relationship despite the large number of studies included. However, the authors point out that the observed preeminence of null associations should be interpreted cautiously because of the low quality of available studies. Likewise, [4] find very little evidence of an effect of community food environments surrounding schools on food purchases and consumption, but did find some evidence of an effect on body weight.

Many systematic review articles have been published attempting to explain this lack of quantitative evidence of the relationship between community food environments and health outcomes. These publications have suggested that the absence of compelling direct evidence is mainly the result of one factor: the insufficient validity and reliability of food environment measurements. [6,7] surveyed peer-reviewed publications from 1990 to 2015 in which food environments were assessed using quantitative approaches. They identified four types of methodologies used: (i) geographic analysis, (ii) sales analysis, (iii) nutrient analysis, and (iv) menu analysis. Only 25% of these studies show any metric evidence (i.e., validity and reliability indices) that validates their quantitative approach for food environments. These instruments are standardized assessment tools, such as the Nutrition Environment Measure Survey (NEMS) [8], which are typically paper-based forms filled out by the subjects themselves (i.e., self-reported) or by a trained observer. In general, these instruments present multiple methodological challenges that limit the understanding of a particular food environment: (i) limited geographical coverage, (ii) high sensitivity to the types of facilities included in the analysis, and (iii) high implementation costs, among others [6,7].

Other approaches that are receiving increasing attention for assessing food environments quantitatively are those methodologies based on geographical information system (GIS) technologies. These methods use the actual locations of the food facilities (i.e., stores, supermarkets) to estimate different measures such as facility density or proximity to the nearest facility [9]. On the basis of these measures, researchers are able to build different definitions of the level and intensity of exposure of a particular individual to a given food environment. Thereby, the GIS-based alternatives solve the problems of traditional methods, which creates a new and important opportunity to finally uncover the actual relationship between food environments and health outcomes, quantitatively [9].

Thanks to the considerable heterogeneity in the use of GIS methods and empirical evidence that utilize these techniques to analyze different food environments, their use has led to an increasing number of null results for the establishment of a robust association between community food environments and other health outcomes such as obesity and sedentarism, among others. In a recent systematic review, [9] conclude that the methodological constraints of using GIS methods center around the lack of validation evidence and standardization of data sources. Generally, information about facilities in community food environments is obtained using either administrative records or commercial sources with no extra quality validation. The resulting poor quality data can lead to uncertainty, bias, and reduced statistical power [10]. In order to boost the potential of GIS-based solutions for studying food environments, developing new validated, standardized, and replicable GIS-based methods are necessary to take advantage of this type of solution and, ultimately, to better understand food environments [9].

In our case, the need for a tool to assess urban environments arose when studying the prevalence of diet inadequacy in children and adolescents aged between 6 and 18 years from educational institutions in the city of Mataró (Catalonia, Spain) [11]. That study demonstrated that adherence to a Mediterranean diet was lower among adolescents and children who had money to spend at school. Because the availability of money is not a risk factor per se if there is no easily accessible unhealthy food, it was decided to study the food environment around schools. Thus, the aim of this paper is to introduce and assess the validity and reliability of a new GIS-based tool called the facility list coder (FLC), developed to meet the above-mentioned need. This tool is based on secondary data, and offers a low-cost, scalable, efficient, and user-friendly way to indirectly identify community nutritional environments.

## 2. Materials and Methods

### 2.1. Case Study Selection

In order to validate the FLC, we used the case of Mataró, a coastal city located near Barcelona (25 km) in Catalonia, Spain. The city has experienced an increase in population in the last 50 years (from 40,407 inhabitants in 1960 to 122,905 in 2010) owing to migration from other parts of Spain and, in recent years, from other nations (mainly from Morocco). The economy of Mataró is mainly based on services (63% of total invoicing) and industry (31%) [12]. The mixture of population and culture is associated with an increased risk of health-related problems such as child overweight and obesity [11]. Among the main determinants of this situation, the food environment around schools stands up. However, the lack of information on the number and type of facilities in this city has obstructed the analysis of the influence of food environment on nutritional outcome [11].

### 2.2. Secondary Data: Introducing the Facility List Coder (FLC)

The Facility List Coder (FLC) is an open source tool developed in Python 3.7 that combines GIS analysis with standard data techniques. In the present text, the term ‘facility’ is used to name any installation, equipment, or place that could be an element of interest when assessing community food environments. Besides other GIS-based solutions, the FLC collects geographical information and facility characteristics from two main GIS search-engines that are available online (Google Maps and Open Street Maps), performing a spatial query around a pre-defined zone around a centroid (e.g., homes or schools), after which information is classified based on the meta-data available for each location based on a comprehensive, multi-language list of key words that allows for the categorization of each facility. These data sets are built utilizing the concept of nodes (or places), which include any geographical objects, such as bridges, street lights, stores, schools, and parks, among others. Besides the geographical location, each place provides different types of information like their description, characteristics, and offers, among others. This information is a combination of self-reported data by users and centrally collected information by each company or organization.

**Figure 1:**
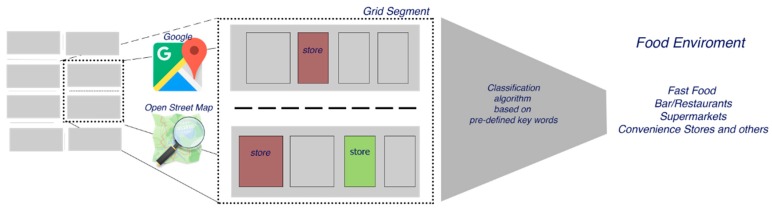
Facility list coder workflow (FLC). The diagram shows the steps performance for the FLC to assess the food environment. For a selected zone in the city map, a spatial query is performed using Google Maps and Open Street Maps, and data on different facilities located in the zone (e.g., food stores) are classified according pre-defined key words, so facilities can be classified in major categories to study the food environment.

The FLC performs a spatial query, retrieving all types of facilities present in a pre-defined zone (e.g., buffer around an interest point or any geographic object). In the case of Google Maps, we used the Application Programming Interface (API) that offers a low-cost and very efficient spatial query. For Open Street Maps, we implement a spatial query taking all nodes that could be classified as facilities. In order to avoid duplicates, FLC performs different techniques based on location, as well as all available meta-data for each location. Once the complete list of facilities is obtained, each facility (e.g., convenience food store, bar, and bakery) is automatically classified using the meta-data available in each data set. On the basis of the classification provided by [13], we built a comprehensive, multi-language list of key words that allows for the categorization of each facility into four types: (i) fast-food restaurants, (ii) bars/restaurants, (iii) supermarkets, and (iv) convenience stores and others. These categories can be modified to fulfill the specific needs of researchers; for example, related to geographical location, multi-lingual search options, or research questions. Although other researchers have used similar categories [13], our pre-defined multi-lingual key word list offers a contribution for researching community food environments within the European context, as empirical studies for Europe often use categories created for the United States, which might incorrectly estimate the particularities of European food traditions. Furthermore, this list can be easily modified and new terms can be incorporated or deleted depending on the needs of the researchers. Finally, taking advantage of the different measures available for GIS, the FLC provides different measures, such as (i) the geographical distance taking into account the road network, in kilometers; (ii) the average time of the walking distance, in minutes; and (iii) the average time of the cycling distance, in minutes. As its main output, the FLC offers a detailed data set for all the classified facilities located around each point of interest. Figure 1 resumes the FLC workflow. Original codes from FLC are available as Supplementary Material.

### 2.3. Street Audits (Physical Verification)

**Figure 2:**
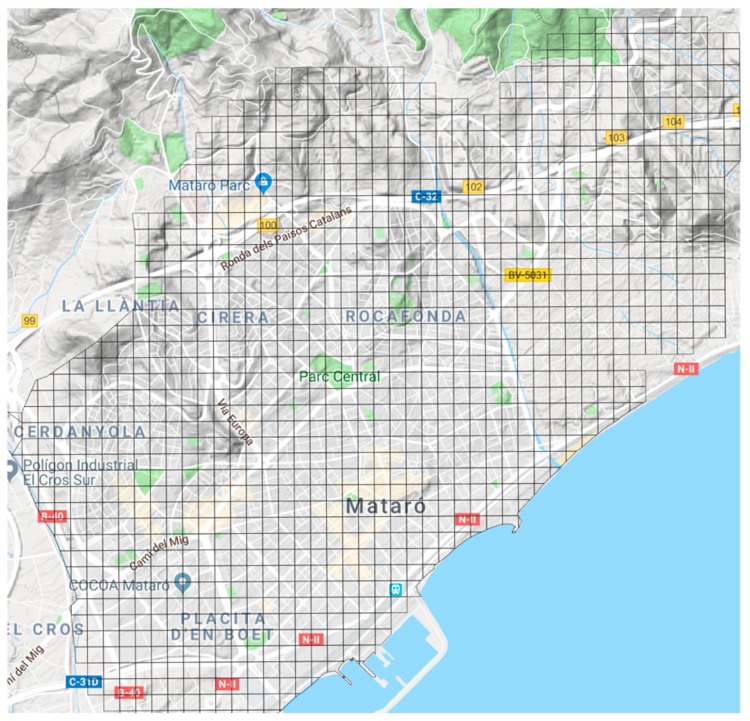
Sampling grids (100 m × 100 m) drawn over the Mataró map and used to sample audit grids.

In order to study the validity and reliability of the output provided by the FLC, we employed a physical verification test (street audits). For the purpose of creating an exogenous unit of analysis, we divided the territory under study into grids of 100 m by 100 m. In total, we created 1375 grids (see Figure 2).

**Figure 3:**
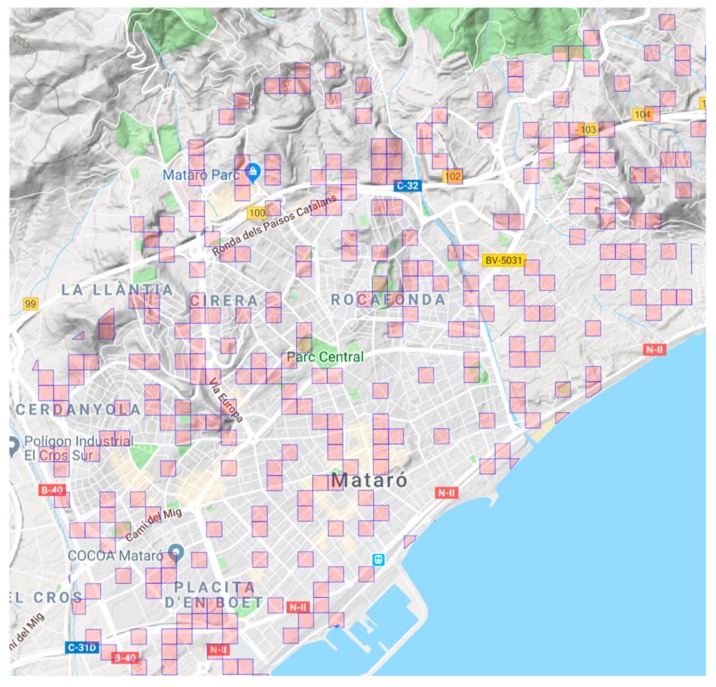
Randomly selected grids (100 m × 100 m) drawn over the Mataró map with the sampled street audits marked in magenta.

On the basis of these grids (“buffer zone” in GIS terminology), we built a simple random sample using a 95% confidence level, with a finite population. In order to estimate the sample size, we used the FLC results to define the expected proportion and variance with a 95% confidence level. In total, 301 grids were randomly chosen (22% of the total). Figure 3 shows the final sample selection.

Two trained people walked the selected grid to record the facilities located along each grid using a tool developed previously with Open Data Kit (https://opendatakit.org/). For each of these facilities, they recorded its name, address, and exact coordinates, and took a picture of each storefront. Finally, on the basis of the classification provided by [13], our team classified each facility into four categories: (i) fast-food restaurants, (ii) bars/restaurants, (iii) supermarkets, and (iv) convenience stores and others. This physical verification was carried out in February of 2018.

The physical verification test allowed us to find three types of facilities: (i) facilities that were found using the FLC, but that were not physically present (false positives); (ii) facilities that exist, but were not identified by the FLC (false negatives); and (iii) those that were identified using both methods.

### 2.4. Statistical Analysis

A descriptive agreement analysis based on the paired t-test and Bland–Altman plot was performed to assess the differences in the number of facilities on each grid obtained using the FLC and those obtained in the street audit, which we consider the “gold standard” method. Whereas the paired t-test allowed us to determinate whether there exists a systematic difference between the two methods, the Bland–Altman plot allowed us to visually identify the agreement pattern by plotting the difference between the two methods on the vertical axis of the diagram with the average of these same methods on the horizontal axis [14]. So, both analyses provide information to study the validity of the FLC.

Then, in order to establish the degree of correlation between the two methods, we used the intra-class correlation coefficient (ICC), widely used for inter-rater reliability analysis. This index is based on McGraw and Wong [15] and there are 10 different forms of the ICC corresponding to different contexts. In our context, as we were interested in assessing the reliability based on the mean of the two methods (i.e., the FLC and field work); we estimated the ICC based on a mean-rating (k = 2), absolute-agreement, two-way mixed-effects model. ICC values less than 0.5 are indicative of poor reliability, values between 0.5 and 0.75 indicate moderate reliability, values between 0.75 and 0.9 indicate good reliability, and values greater than 0.90 indicate excellent reliability [16]. Moreover, to control for the potential bias of having a lot of pairs of zeroes that may artificially inflate the apparent reliability, we used Krippendorff’s alpha reliability estimate, which is an alternative to estimate reliability, allowing for controlling for the presence of zeros. All analyses were performed using R.

## 3. Results

After applying the facility list coder (FLC) to Mataró using 100 m × 100 m grids, we identified 935 facilities. According to our results, the most common type of facility was “bars/restaurants”, representing 25.8% of all identified facilities, followed by “fast-food restaurants” with 18.9%.

**Figure 4:**
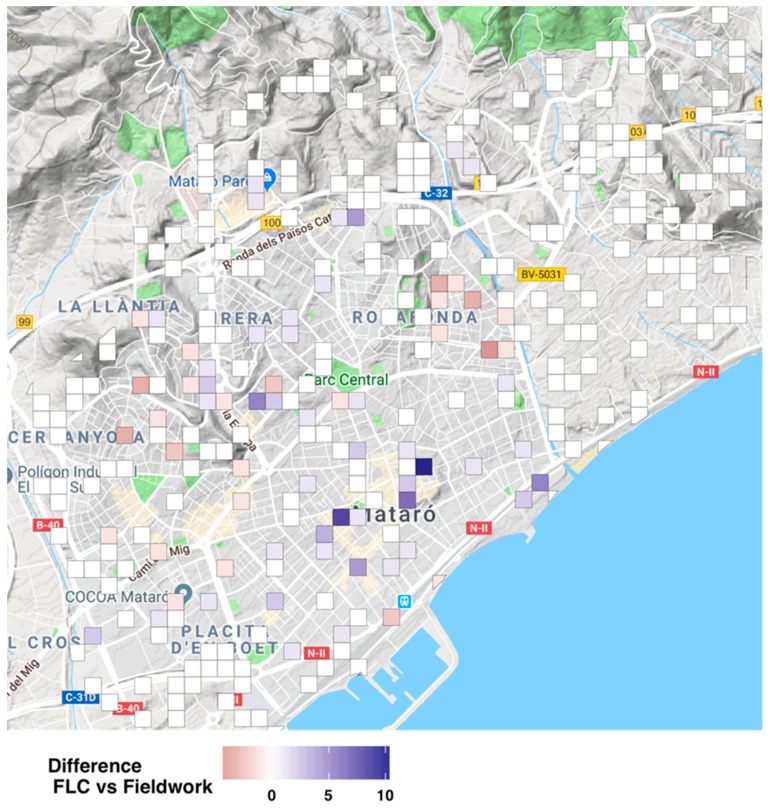
Spatial distribution of the difference between the FLC and the street audit (field work). The map shows the difference between facilities found using the FLC and street audit at the randomly selected grids; purple color stands for false positives (i.e., FLC larger than field work), pink color stands for false negatives (i.e., FLC smaller than field work), and white color stands for a perfect match between the two methods.

Figure 4 shows an overview of the results from comparing the field work and the FLC results; purple color stands for false positives (i.e., FLC larger than field work), pink color stands for false negatives (i.e., FLC smaller than field work), and white color stands for a perfect match between the two methods. Overall, we found that the FLC performed well compared with the street audit. In fact, for 78% of the selected streets, we found the exact same number of facilities through both methods. Moreover, when allowing for a tolerance rate of just one facility (i.e., both methods could differ in +/– one facility), this agreement rate rose to 92.4%. Likewise, we found around 14% of false positives (those facilities that were found using the FLC, but that were not physically present) and 8% of false negatives (facilities that exist, but were not identified by the FLC). The paired t-test statistics is 0.976 with 573 degrees of freedom (*p*-value = 0.329). Hence, there was no evidence of a systematic difference between the results from the FLC and the field work. In both methods, we found that these methods are valid as they are able to properly represent the universe of facility in our case of study.

**Figure 5:**
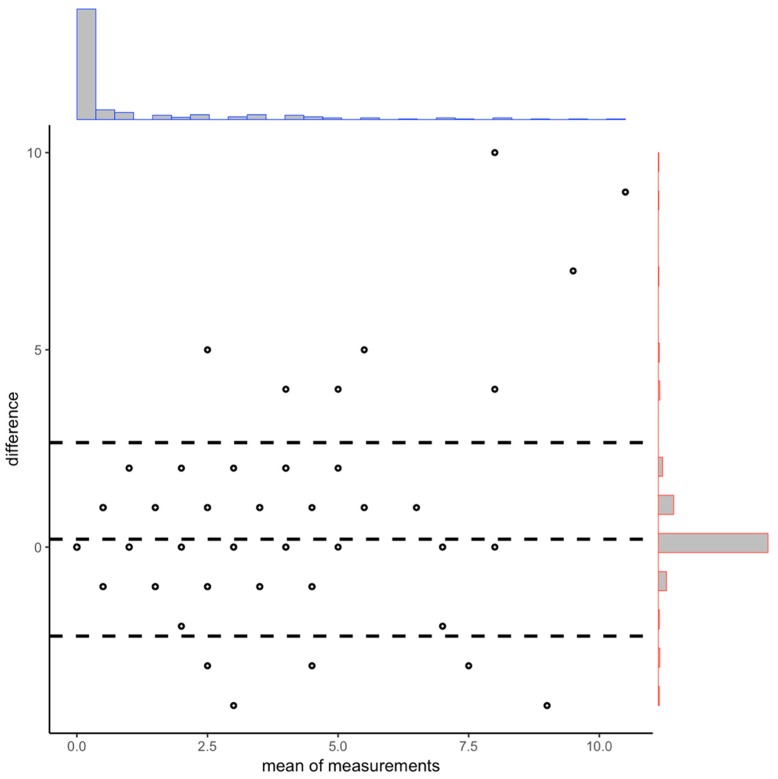
Bland and Altman diagram for the comparison of results obtained with FLC versus street audit.

The Bland–Altman diagram provides a first glance at the pattern of agreement between the two methods (see Figure 5). As we pointed out, we observed a high level of agreement between the two methods for the total number of facilities per grid. However, we did find an important disagreement between the FLC and the field work results on those grids with the two largest numbers of facilities [9,10]. After checking manually, we found that these differences were mainly because of how local food markets were counted; whereas the field work treated the market as a single facility, the FLC coded all the facilities that were located within the markets individually, which is obviously more convenient.

The ICC estimates and their 95% confidence intervals for the overall sample indicated that the level of reliability is in the range of good to excellent. When we corrected the data for the local markets, our results got an excellent reliability index using the ICC, which were in any case always above 0.9. Once we take into account the zero bias (Krippendorff’s alpha) results are still showing a high degree of reliability (see Table 1).

When we compared the ICC results by type of facility, we found good to excellent results for all types of facilities (Table 2). The ICC for bars/restaurants was excellent (0.92), followed by fast-food restaurants (0.86) and supermarkets (0.82). The worst performance was found within the category of convenience stores and others, where the ICC was 0.76, which is still acceptable according to the criteria mentioned above. These results suggest that the automatic classification of facilities performed by the FLC is consistent with the classification performed by direct observation. As before, Krippendorff’s alpha confirms our results as well as the false positive rate.

## 4. Discussion

Assessing food environments using GIS-based approaches offers an ample methodological range of possibilities that can overcome the most traditional challenge to finding quantitative evidence for the relationship between food environments and health outcomes [3,4]. This study sought to validate a new tool called the facility list coder (FLC), which allows for evaluating community food environments, using secondary data obtained from the two most traditional geographical online search-engines: Google Maps and Open Street Maps. We used the case of Mataró (Spain) to validate this tool, comparing the automatic facility classification provided by the FLC with the ‘gold-standard’ obtained using physical direct verification. Our results indicate that the FLC has good to excellent reliability with respe

+ct to the street audit—hence, the FLC provides an excellent source of information for studying food environments.

The FLC fulfills the five main requirements suggested by [10] for validating a GIS-based approach to food environments: (i) food outlet data, (ii) extracting food outlets, (iii) defining food outlet constructs, (iv) geocoding methods, and (v) access metrics. Information for GIS search-engines is centrally managed by each company, yet they are often updated by users (food outlet data). As a result of this spatial query, we retrieved all types of facilities present in a pre-defined grids. Because a spatial query is based on a pre-defined location, including particular search terms (extracting food outlets) is not necessary. Once the complete list of facilities is retrieved, they are classified using an exhaustive list of key words following [13]. Likewise, because other meta-data are also collected, information can be easily verified (defining food outlet constructs). Because the information is already geocoded, no further geocoding methods are needed (geocoding methods). Finally, taking advantage of the pre-defined GIS search-engine algorithms, the FLC provides different measurements of distances, such as network distance and walking distance, among others (access metrics).

One of the main concerns related to measuring food environments using secondary data sources is the lack of adequate evidence of their validity and reliability. Many researchers have highlighted this fact as being one of the main limitations of their studies [4,10,17]. Very often, researchers use a facility census or facility lists as the main source of information for assessing food environments. These data are mainly collected for official or commercial purposes and often present several limitations related to geographical location and update, which leads to high heterogeneity in the data quality among different sources. [18] compared two different data sources for food outlets in the United States and found that, depending on the data source selected, the level of statistical significance of the association between neighborhood racial and socioeconomic characteristics and food/alcohol facility density varies. This empirical problem is mainly the result of the large difference between the two data sources and it points out the importance of data validation in avoiding bias. In order to overcome these challenges, researchers should compare to a ‘gold standard’ like physical verification (street audits) [10,19]. Using this approach, [19] validated the two main data sources for the United Kingdom through street audit verification, concluding that these two secondary data sets provide a good view of the actual state of food environments. Nonetheless, utilizing a ‘gold standard’ is not always possible as it is often demanding financially as well as time-wise. In these cases, the FLC contributes good to excellent reliability and might offer a complementary data source for researchers so they can have a benchmark with which to validate or complement their initial results using the additional information for food environments.

Sociodemographic dimensions could trigger effects of any food environment on health outcomes [2]. Former studies have shown that low-income families are more likely to be affected by their surrounding food environment [4,20]. Hence, assessing validated and standardized measures of food environments can be difficult—for example, low-income areas pose an empirical challenge as administrative data are often low-quality or simply non-existent. In these cases, the FLC can be used as the main source of information to identify community food environments in cases where researchers or practitioners have a limited budget, or the area of study makes it impossible to utilize other intensive techniques such as a facility census. Furthermore, even considering that the quality of data provided by this GIS systems is not homogenous for all countries, this GIS information has worldwide coverage, so the FLC might provide a proxy for the food environment in places where the coverage and the data quality is good, but an official facility census or directory does not exist or is not available, as in our case. However, reproducibility of the FLC in other globe locations should be checked.

As [10] have mentioned, the GIS-based tool has limitations of which users need to be aware. As the FLC uses the most popular GIS search-engines to assess food environments, it can be a source of measurement error, as information could be either centrally generated by the search-engines or self-reported by users. Despite this, all the information available is verified and standardized to guarantee good quality control [21,22]. The fact that part of the information is self-reported by users might lead to the following potential limitations: (i) the FLC might underestimate the food environment in places with a small amount of GIS information and (ii) the FLC might misallocate facilities in locations where no further information is available. Although it is impossible to rule these biases out completely, other researchers [23] have evidenced the validity and good quality of this information. We confirm this in our research.

Another concern is the automatic facility classification into pre-defined categories. [13] present a literature review that delineates how to create a detailed guide for developing classifications of food environments. They conclude that it is not possible to provide only one classification that can be applied in any context. Therefore, we opted for a simplistic and conservative classification adapted to the Spanish context for four categories: (i) fast-food restaurants, (ii) bars/restaurants, (iii) supermarkets, and (iv) convenience stores and others. As [19] claim, although this general classification does not take into account food provision within individual outlets nor other factors that may influence purchasing decisions, such as pricing and preferences, it provides an opportunity for a baseline analysis and it presents a possibility for future large-scale research projects [19].

The FLC is not the only tool that can be used to assess food environments using common online search-engines like Google Maps. The SPOTLIGHT-Virtual Audit Tool (S-VAT) uses the street views provided by Google Earth to develop a desk-based assessment of community food environments [21,24]. This tool was derived from a large European Union-funded project and was developed to identify and compare environmental characteristics in European neighborhoods. Along with the street images, researchers are provided with a pre-defined form through which they can virtually ‘audit’ each street segment by segment. As a result, on the basis of their storefronts, a list is compiled of all the facilities, as well as other characteristics such as walkability, cycling-related infrastructure, and public transport, among others. [21] found that S-VAT was a highly reliable tool for classifying food environments using street view images.

The FLC differs from the S-VAT in many ways. First, the FLC focuses only on determining the characteristics of each food environment through building a classification system of facilities in pre-defined categories, while the S-VAT only relies on the storefront image, which can lead to important misclassifications. Second, unlike the S-VAT, the results from the FLC provide a list of all the classified facilities, which allows for properly classifying every food environment. Third, as the S-VAT is based on the visual audit of each street, it is more difficult to collect meta-data or characteristics of each facility. The FLC gathers all the information available for each store (e.g., type, images, and opening hours), which provides a better understanding of the food environment. Therefore, the FLC and S-VAT, rather than being equivalent tools, complement one another.

## 5. Conclusions

To conclude, the FLC is a valid and reliable tool for evaluating community food environments in our case of study. This result is building evidence of the validity of using a GIS-based solution like FLC to evaluate food environments, which can be used either as a validation of other secondary data or as a main source of information. The FLC uses the most popular data sources (i.e., Google Maps and Open Street Maps) to identify the facilities present around a given location (e.g., school, hospital, and university). As a result, researchers can have access to a comprehensive list of facilities around any location of interest, allowing for a more detailed investigation that informs key research questions about the influence of food environments on multiple public health outcomes, such as obesity, sedentarism, and dietary patterns, among others. In sum, the FLC offers a new, low-cost, scalable, efficient, and user-friendly tool to assess food environments, and it can be implemented in different types of research projects that want to include food environments as a dimension of analysis.

## Figures and Tables

**Table 1:** Intra-class correlation coefficients (ICCs) calculated using a mean-rating (k = 2), absolute-agreement, two-way mixed-effects model. df, degrees of freedom.

	Interclass Correlation						Krippendorff’s Alpha		False Positive Rate
	ICC index	95% Confidence Interval		F Test with True Value 0					
		Lower Bound	Upper Bound	Value	Degree of freedom 1	Degree of freedom 2	Significance		
Overerall Sample	0.898	0.872	0.919	9.94	300	287	0.000	0.875	14%
Overall sample after correcting for markets	0.933	0.916	0.946	14.9	297	296	0.000	0.870	13%

**Table 2:** Intra-class correlation coefficients calculated using a mean-rating (k = 2), absolute-agreement, two-way mixed-effects model. Sample after correcting for markets.

	Interclass Correlation	95% Confidence Interval		F Test With True Value 0				Krippendorff’s Alpha	False Positive Rate
		Lower Bound	Upper Bound	Value	Degree of freedom 1	Degree of freedom 2	Significance		
Fast Food	0.861	0.825	0.889	7.18	297	298	0.000	0.770	2%
Bar/Restaurants	0.926	0.907	0.941	13.5	296	297	0.000	0.840	11%
Supermarkets	0.827	0.780	0.864	5.96	297	237	0.000	0.810	5%
Convenience Stores and others	0.764	0.703	0.813	4.30	297	282	0.000	0.587	2%

## Supplementary Materials

The original codes are available at https://github.com/jcmunozmora/facilitylistcoder.git.
